# Plasmablastic Lymphoma or Plasmablastic Myeloma: A Case of Post-Transplant Lymphoproliferative Disorder

**DOI:** 10.1155/2021/4354941

**Published:** 2021-09-27

**Authors:** Poornima Ramadas, Michael Williams, David B. Duggan

**Affiliations:** ^1^SUNY Upstate Medical University, 750 East Adams Street, Syracuse, New York 13210, USA; ^2^LSU Health Sciences Center, 1501 Kings Hwy, Shreveport, LA 71103, USA

## Abstract

Plasmablastic lymphomas and plasmablastic myelomas are malignancies with overlapping clinical and pathological features which pose a diagnostic dilemma and are known to be aggressive with a poor outcome. CD38 is a common immunophenotypic maker for both these malignancies and provides a rationale for using daratumumab-based regimes. We describe a 57-year-old male with a history of end-stage renal disease who underwent a deceased-donor renal transplant maintained on chronic immunosuppression who presented with ascites and was found to have abdominal adenopathy and a lytic lesion in the humerus and diagnosed with a post-transplant lymphoproliferative disorder with features intermediate between plasmablastic lymphoma and plasmablastic myeloma. The patient was subsequently treated with a daratumumab-based regime with an excellent response. This case highlights a rare scenario that poses a diagnostic and therapeutic challenge. As there is no standard of care for the treatment of both these malignancies, this case report also describes the use of daratumumab with a good long-term outcome, especially when the pathological distinction between the two entities is difficult.

## 1. Introduction

Post-transplant lymphoproliferative disorder (PTLD) is the most common malignancy that occurs in recipients of solid organ transplants and occurs because of immunosuppression (IS) [1]. Monomorphic PTLD includes lymphomas and plasma cell neoplasms. Plasmablastic lymphoma (PBL) is a rare aggressive lymphoma that is usually associated with IS with no definite standard of care and a poor outcome [2]. Plasmablastic myeloma (PBM) is a rare morphological variant of plasma cell neoplasm which is also known to be extremely aggressive with poor survival [3]. Both these malignancies have overlapping clinical and pathological features which pose a diagnostic dilemma [4]. In addition, treatment options are not well defined for either of these and are decided based on institutional/physician preferences and the clinical scenario. CD38 is a common immunophenotypic marker for both these diseases. This provides a good rationale for using daratumumab-based regimes in management, especially when the pathological distinction between the two entities is difficult.

In this case report, we describe a patient with PTLD with features intermediate between PBL and PBM, with clinical features more consistent with PBL, and with an excellent response to a daratumumab-based regimen.

## 2. Case Presentation

**Figure 1:**
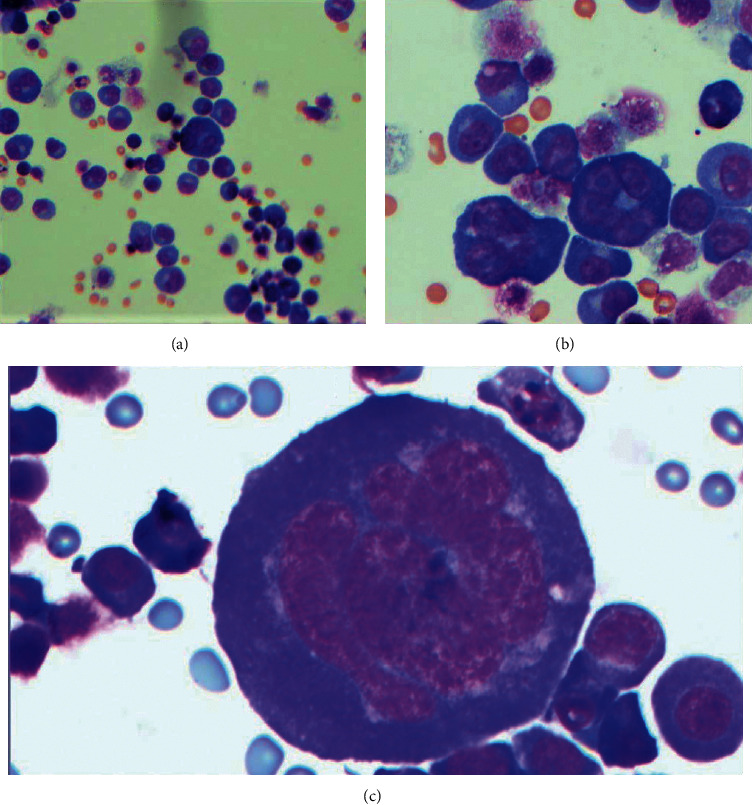
Ascitic fluid cytology showing large immature cells, some of which are multinucleated and admixed with smaller mature looking plasma cells. (a) 200X magnification. (b) 500X magnification. (c) 500X magnification showing large cell with multilobed nucleus.

**Figure 2:**
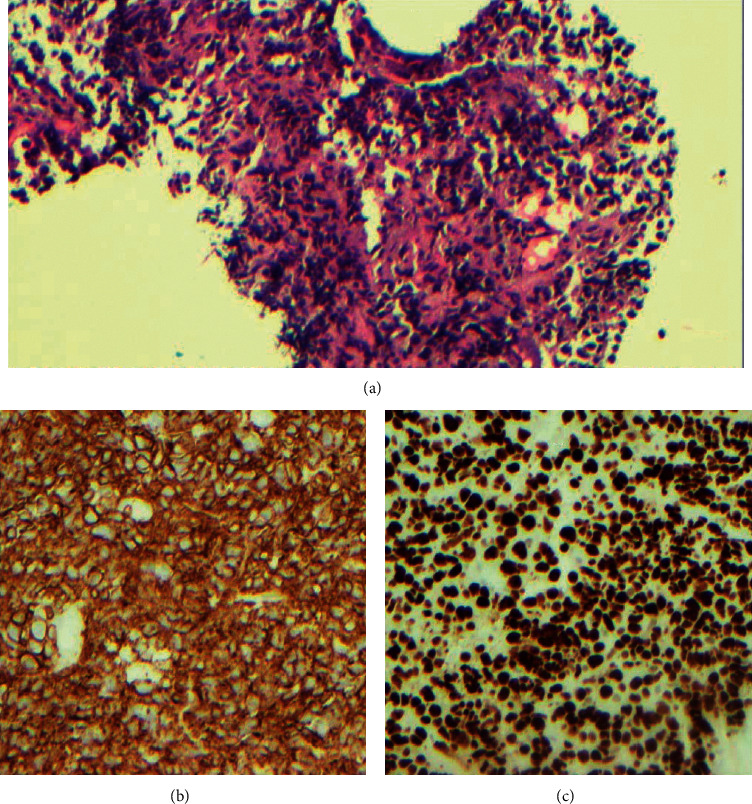
Lymph node biopsy. (a) 4X magnification showing infiltration of the node with abnormal uniform looking cells. (b) 200X magnification for Ki67 highlighting >90% of cells. (c) 200X magnification of CD138 stain.

A 57-year-old male who underwent a deceased-donor renal transplant in 1993, maintained on chronic IS with tacrolimus, mycophenolate mofetil, and prednisone, presented with abdominal distention for 2 weeks. He had a history of end-stage renal disease due to hypertension, renal cell carcinoma status post-right-sided nephrectomy, andright lower extremity deep vein thrombosis (DVT) in 2012 and left lower extremity DVT in 2013 and was on anticoagulation. Initial labs showed hemoglobin of 11.4 g/dL (similar to baseline), WBC count of 16,700/uL, platelets of 313,000/uL, with creatinine of 3.13 mg/dL (baseline around 2.5 mg/dL), and corrected calcium of 9.3 mg/dL. CT abdomen pelvis showed a moderate to a large amount of ascites with multiple small, enlarged lymph nodes within the abdomen and pelvis along with the nodularity of the mesentery within the anterior abdomen. He underwent paracentesis, and cytology was reported as plasma cell neoplasm. Immunophenotype showed bright positivity for CD45, moderate positivity for CD138, lambda-restriction, and was also positive for CD19, CD38, and CD56, and negative for CD20 (Figure 1). Cytogenetics showed 5% of nuclei with three copies of 1q and 2 copies of 1p with no evidence for gains or losses of chromosome 5; deletions of 13q14.2, 14q32.3 (IgH), or 17p13 (TP53); or an IgH rearrangement. Given cytology findings, serum protein electrophoresis and immunofixation were obtained which showed a small IgA lambda paraprotein of 570 mg/dL and total IgA of 740 mg/dL. Urine protein electrophoresis was normal. Serum free light chains showed lambda light chains of 163 mg/L, kappa light chains of 58 mg/L, and a normal kappa lambda ratio of 0.36. Uric acid was 11.1 mg/dL and LDH was 634 U/L. CT thorax and soft tissue neck showed small to moderate bilateral pleural effusions and a 1.7 × 3.3 cm lymph node in the superior diaphragmatic region. Over the next few days, he was noted to have worsening renal failure with oliguria and underwent biopsy of the transplanted kidney, and pathology was suggestive of calcineurin inhibitor toxicity with no evidence of rejection or light chain deposition. He also underwent biopsy of the node in the diaphragmatic region, and pathology was consistent with plasma cell myeloma with anaplastic morphology with an immunophenotype like the ascitic fluid (Figure 2). Bone marrow biopsy showed normocellular marrow with no evidence of plasma cell neoplasm. He started having right arm pain and underwent a CT scan which showed intramedullary soft tissue density within the mid-humeral diaphysis with a central lytic lesion and associated areas of cortical breakthrough. He was diagnosed with PTLD with intermediate features between PBL and PBM.

Immunosuppression was tapered and stopped per recommendations from transplant nephrology. He was transferred to hematology service and started on cyclophosphamide, bortezomib, and dexamethasone along with daratumumab. He also started having right foot pain, and CT scan showed midfoot patchy demineralization and mild fragmentation, possibly an acute lytic process. He had ascitic fluid accumulation again and underwent repeat paracentesis 11 days after the initial procedure. He was continued on weekly cyclophosphamide, bortezomib, dexamethasone, and daratumumab. He underwent radiation treatment to the right arm to a dose of 2400 cGy in 6 fractions and the right ankle to a dose of 2400 cGy in 6 fractions. Ascitic fluid accumulation started improving on treatment. He received 4 weekly treatments during the initial inpatient stay and was discharged once stable. He was readmitted 6 days later with a pathologic fracture of the right humerus for which he underwent open reduction internal fixation. He resumed treatment as an outpatient. Cytoxan, bortezomib, and dexamethasone (CyBorD) were continued weekly (days 1, 8, 15, and 22 of 28-day cycles), and daratumumab was given per the standard de-escalation schedule (weekly for 8 doses followed by 2 weekly doses). After around 5 months since the initial diagnosis, he underwent repeat CT scans which showed significant overall improvement in the abdominal ascites and mesenteric lymphadenopathy. He also underwent a SPECT-CT which only showed some uptake in the right humerus but no other areas of uptake. He started developing severe neuropathy, and bortezomib was held from cycle 6 day 15. In addition, he started developing generalized bone pain with no etiology on imaging, and this was thought to be related to daratumumab. He completed a total of 6 cycles of treatment, which included 14 doses of daratumumab. Given multiple adverse effects and good response to treatment, the decision was made to stop treatment and monitor the patient closely. He is currently 18 months post his initial diagnosis and continues to be in remission. It was decided not to resume immunosuppression for renal transplant, and he has been initiated on hemodialysis.

## 3. Discussion

PBL is a rare subtype of diffuse large B-cell lymphoma (DLBCL) with plasmacytic differentiation. Around 70% is associated with HIV positivity, and the remaining 30% is seen in HIV-negative patients, of which iatrogenic immunosuppression is a common etiology. It also has a strong association with Epstein–Barr virus (EBV) infection and may be positive in around 80% of the cases [5]. Immunophenotype shows an absent expression of CD45, CD20, CD79a, and PAX5 and strong expression of plasma cell markers, CD38, CD138, and MUM1, along with high Ki67 expression [6]. Rearrangements in MYC oncogene were seen in around 50% of the cases and more often seen in EBV positive tumors [7]. The oral cavity and gastrointestinal tract are the most commonly involved sites in both HIV-positive and HIV-negative patients, but any other site including CNS and bone marrow can be involved [5]. Treatment involves multiagent chemotherapy, and intensive regimens like infusional EPOCH, HyperCVAD, or CODOX-M/IVAC are recommended over cyclophosphamide, doxorubicin, vincristine, and prednisone (CHOP), though it is unclear if these regimes offer a survival benefit [8]. Bortezomib and lenalidomide have also been studied, especially in refractory cases [9]. Though an aggressive disease, treatment with chemotherapy improves survival as reported in a SEER database analysis published in August 2020 which showed a 5-year overall survival of 52.8% in treated PBL patients [10]. The presence of a MYC rearrangement has been reported to have a poor prognosis [7].

PBM is a subtype of multiple myeloma (MM) comprising plasmablasts, which are immature plasma cells with clear round nuclei, nucleoli, and small amounts of cytoplasm. Immunophenotype shows positive CD38 and CD138 along with light chain restriction [4]. In a population-based study, patients with plasmablastic morphology had unfavorable clinical features, extensive marrow infiltration, and high Ki67 but did not correlate with poor-risk cytogenetics [11]. Treatment involves myeloma regimes. PBM is also known to be an independent predictor of poor survival after autologous stem cell transplantation [12].

The distinction between PBL and PBM can be a diagnostic dilemma. The immunophenotype of these is remarkably similar, and all patients with PBL and PBM show positivity for CD38, CD138, and MUM1 with high Ki67 and are negative for CD20. CD56 can be positive in PBM, and EBER can be positive in PBL [13]. A study published in March 2017 identified the clinical and pathological features between the two entities and proposed a diagnostic algorithm [14]. The truly borderline cases also pose a challenge in making a treatment decision. Our patient had intermediate features between PBL and PBM. The presence of a paraprotein with lambda restriction on pathology, lytic lesion, and negativity for EBER supported PBM. However, the clinical presentation with the extramedullary disease with nodal involvement and ascites with no evidence of marrow involvement and the mixed immunophenotypic features supported PBL.

Daratumumab is an IgG1*κ* human monoclonal antibody against CD38 and inhibits the growth of tumor cells with CD38 expression by inducing apoptosis. Daratumumab is well studied in multiple myeloma although the data with PBM have only been reported in case reports [15]. In PBL, a feasibility multicenter study has been proposed, but most of the studies which reported the use of daratumumab are case reports and have been used in relapsed refractory disease [16, 17]. Given that CD38 is a common immunophenotypic marker for both these diseases, the use of daratumumab-based regimes can be considered in borderline cases. As our patient had intermediate features, the decision was made to use daratumumab up front, and given his overall functional status, we did not believe that he could tolerate a more intensive lymphoma regime.

In conclusion, this case report highlights a rare scenario of PTLD where the distinction between PBL and PBM was a diagnostic challenge and describes the use of daratumumab with good long-term outcome. Daratumumab should be considered in the initial treatment of CD38-positive PTLD when the pathological features overlap. Further studies are needed in exploring the use of daratumumab in PBL.

## Data Availability

No data were used to support the study other than reported.
